# Leaf Segmentation and Tracking in *Arabidopsis thaliana* Combined to an Organ-Scale Plant Model for Genotypic Differentiation

**DOI:** 10.3389/fpls.2016.02057

**Published:** 2017-01-11

**Authors:** Gautier Viaud, Olivier Loudet, Paul-Henry Cournède

**Affiliations:** ^1^Laboratory MICS, CentraleSupélec, University of Paris-SaclayChâtenay-Malabry, France; ^2^Institut Jean-Pierre Bourgin, INRA, AgroParisTech, CNRS, Université Paris-SaclayVersailles, France

**Keywords:** segmentation, tracking, genotypic, differentiation, Arabidopsis, leaf, area, phyllotaxy

## Abstract

A promising method for characterizing the phenotype of a plant as an interaction between its genotype and its environment is to use refined organ-scale plant growth models that use the observation of architectural traits, such as leaf area, containing a lot of information on the whole history of the functioning of the plant. The Phenoscope, a high-throughput automated platform, allowed the acquisition of zenithal images of *Arabidopsis thaliana* over twenty one days for 4 different genotypes. A novel image processing algorithm involving both segmentation and tracking of the plant leaves allows to extract areas of the latter. First, all the images in the series are segmented independently using a watershed-based approach. A second step based on ellipsoid-shaped leaves is then applied on the segments found to refine the segmentation. Taking into account all the segments at every time, the whole history of each leaf is reconstructed by choosing recursively through time the most probable segment achieving the best score, computed using some characteristics of the segment such as its orientation, its distance to the plant mass center and its area. These results are compared to manually extracted segments, showing a very good accordance in leaf rank and that they therefore provide low-biased data in large quantity for leaf areas. Such data can therefore be exploited to design an organ-scale plant model adapted from the existing GreenLab model for *A. thaliana* and subsequently parameterize it. This calibration of the model parameters should pave the way for differentiation between the Arabidopsis genotypes.

## 1. Introduction

In order to predict plant phenotypic performance, statistical models are usually built based on linear mixed-effect models for integrative variables (Bustos-Korts et al., 2016). Their strength is that they take advantage of large repetitions of trials in very diverse environmental conditions since they necessitate only restricted amount of data, but they offer poor perspectives in terms of interpretation and extrapolation.

On the contrary, since they rely on the mechanistic description of growth processes, plant growth models have opened promising perspectives for the description and prediction of genotype by environment interactions. Mathematically speaking, if we consider the system of interest as the plant in its environment (or a population of plants, or a specific part of the plant for models at smaller scales) plant growth models could formally be represented in the very generic following form:

(1) Y=f(θ,E)

where:

- *Y* represents all the phenotypic traits of interest, and is generally a real-valued function of space and time.
- *f* represents the functional equations (usually dynamical, see for example the description of plant growth models as dynamic state space models and hidden Markov models in Cournède et al., 2013).
- θ represents all the parameters of the model. Some of them are of biophysical relevance but some are only empirical parameters (parameters of empirical descriptive functions). As we will detail later, the estimation of these parameters is a key issue in plant growth modeling.
- *E* represents all the external variables for the system, which mostly corresponds to the environmental variables. At the global scale, the main variables generally correspond to radiation, temperature, potential evapo-transpiration, soil content in water and nutriments. The agricultural, horticultural, forestry practice can also be represented in the environmental variables.

Models differ with respect to the phenomenon of interest and the studied species.

For a given species and a given model, the parameters should ideally be able to characterize genotypes. As stated by Tardieu (2003), the application associating its model parameter vector to each genotype should be injective. In such an ideal situation, we could imagine very concrete applications: for instance, for a given environment *E*, compare the performances of two genotypes characterized by two different parameter sets θ_1_ and θ_2_. Conversely, if the parameter set is stable for one genotype in a large range of environmental conditions, we can optimize some traits of interest with respect to the environmental conditions (see examples for maize Qi et al., 2010, sunflower Lecoeur et al., 2011 or peach Quilot-Turion et al., 2012), leading to potential decision aid tools. One example would be the optimization of water supply under logistic and availability constraints Wu et al. (2012).

If the ecophysiological parameters characterize a given genotype, then we could also imagine to decompose the genetic variation of model parameters into individual quantitative trait loci, or conversely to design a predictive model determining this parameter set from the plant genetics, that is to say to write θ = *H*(*G*) where *G* represent the genetic sequence of the individual plant, either the genomic sequence or a representation of it with quantitative trait loci markers (see for example Quilot et al., 2005; Hammer et al., 2006; Letort et al., 2008; Xu et al., 2011; Reymond et al., 2003; Des Marais et al., 2016).

As described by Yin and Struik (2010) or Baldazzi et al. (2016), the tendency is to complicate the mechanistic description of biophysical processes, by linking ecophysiology to omics sciences as an attempt to fully comprehend the regulatory networks from which plant robustness and plasticity is supposed to emerge (Hirai et al., 2004) whilst the related robustness appears to be difficult to achieve at the cell or tissue level. The modeling of plant growth and development lends itself to such an integrative approach. Several models for various component systems of plants are constantly developed (Hodgman et al., 2009). However, the road is still long to achieve such an ambitious objective, resulting in a predictive model from the genes to the whole plant phenotype in a large range of environmental conditions. The more complex the models, the more troublesome their parameterization and the assessment of the estimate uncertainty (Ford and Kennedy, 2011), specifically due to costly experimentations and the large number of unknown parameters to consider. Likewise, local environmental conditions (in terms of climatic and soil variables, as well as biotic stresses) and initial conditions in specific fields are also very delicate to characterize. Consequently, the propagation of uncertainties and errors, which are related to parameters and inputs of these dynamic models, may result in poor prediction of the plant-environment interaction in real situations.

A good compromise between mechanistic description of plant growth processes and the level of details in the data necessary for their parameterization has recently emerged with a new paradigm for plant ecophysiological modeling, namely functional-structural plant modeling (see Vos et al., 2009). It combines the ecophysiology of plant growth to its architectural development. One of their fundamental properties is that their parameterization does not rely on the same type of information as classical ecophysiological models: architectural traits have the property to integrate the whole history of plant functioning, and a large information (in the Fisher sense) on model parameters can be inferred from the observation of the architectural traits. The key point that we aim at taking advantage of in this paper is that architectural traits can potentially be measured efficiently by automatic image analysis in high-throughput phenotyping platforms. These have recently gained increasing interest, both in fields (Araus and Cairns, 2014) and laboratories (Tisné et al., 2013), thanks to their capacity to automatically measure many morphological and physiological traits for a large number of plant genotypes in various environmental conditions. However, although these measurements are potentially very detailed in time, they usually concern integrative traits (masses, total leaf area, height, etc.) and are again classically analyzed with descriptive statistical (multifactorial) models (see for example Granier and Vile, 2014).

Our objective in this paper is dual: first, we will propose an image analysis methodology allowing to dynamically monitor surface areas of every individual leaves in *Arabidopsis thaliana* phenotypes and, second, we will show how these architectural data can be used to parameterize a functional-structural model of Arabidopsis growth with the objective of genotypic differentiation. The material and methods section deals with the phenotyping data produced by the Phenoscope platform Tisné et al. (2013), the image analysis methodology and the functional-structural model developed for *A. thaliana*. The Phenoscope platform is first described (section 2.1) and several traits of its output images analyzed (section 2.2) for further use in the image processing methodology. The latter relies on two main steps, segmentation (section 2.3) and tracking (section 2.4). The segmentation part has already been studied Scharr et al. (2016), and the method we developed was largely inspired by Apelt et al. (2015). However, most studies only consider static images and are not interested in the dynamic monitoring of leaf growth, which raises non-trivial problems in tracking. We also propose an adaptation of the GreenLab model (Yan et al., 2004), (Christophe et al., 2008) for the first stage of Arabidopsis growth (section 2.5). The results of the dynamic monitoring of individual leaf surface areas are presented for 4 different genotypes in section 3.1, and are then used to parameterize the GreenLab model with statistical model inversion techniques in section 3.2. These results and further perspectives are discussed in section 4.

## 2. Materials and methods

### 2.1. Data acquisition

Images of *A. thaliana* were acquired using the Phenoscope, an automated phenotyping platform, whose full description can be found in (Tisné et al., 2013). It is made of an aluminum table on a steel structure and allows the simultaneous growth of 735 plants in individual pots that are displaced along guiding rails across the table to ensure that all plants are grown in the same environmental conditions on average. The Phenoscope comprises two stations: a watering station where each pot, when placed over it, is weighed and watered according to instructions with a specified nutrient solution, and an imaging station that captures zenithal images of the plant placed under the digital camera. The Phenoscope is equipped with its own image processing scripts, Phenospeed, that outputs images where the background and leaves from neighboring plants have been removed to keep only the main rosette with red, green and blue color components. These images have width *n* = 1624 pixels and height *m* = 1232 pixels and 1 cm^2^ is considered equal to 28,900 pixels. Phenospeed automatically computes the total projected rosette area (in cm^2^). It cannot, however, computes the individual leaf areas necessary to exploit an organ-scale plant model.

**Figure 1:**
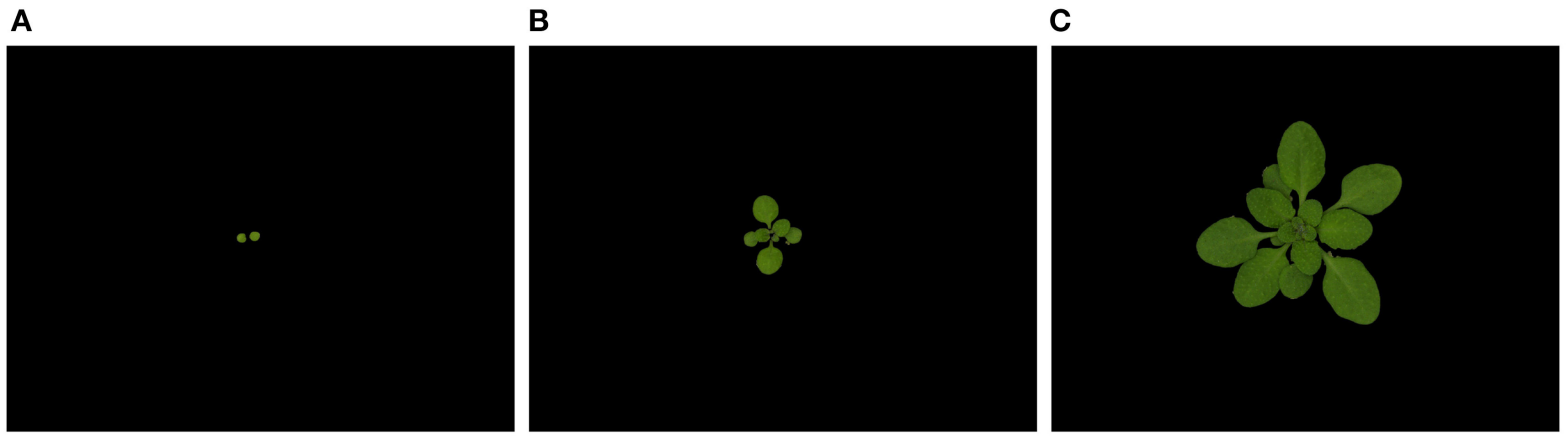
**Images output by the Phenoscope software on different days (after installation on the Phenoscope which takes place 8 days after sowing) for genotype Bur**. **(A)** Day 1. **(B)** Day 11. **(C)** Day 21.

The dataset considered in this article consists of a series of *T* = 21 images for one plant of each of the 4 genotypes Burren (Bur), Columbia (Col), Shahdara (Sha), and Tsushima (Tsu). The plants were all grown in the same environmental conditions. The photoperiod was of 8 h, with a radiation of 350µmol m^−2^ s^−1^. The temperature was set to 21°C during the day and 18°C at night. The hygrometry was maintained constant at 65%RH. The series is composed of images taken on consecutive days from the 9th day after sowing (the day when the plants are installed on the robot) to the 29th day after sowing although, for the sake of clarity, the days of the image series will be identified from 1 to 21 in the following. On day 1 (from installation on the robot), the plants already have fully opened cotyledons (denoted as leaves 1 and 2). It should be noted that, for the sake of clarity, we have numbered leaves including the 2 cotyledons, so that the first true leaf is actually leaf 3. Images for the Bur genotype are presented in Figure 1 on three different days.

### 2.2. Data analysis

To better understand the dynamics of the whole plant, a series of measurements was performed on each image for the 4 genotypes available. Images are considered as elements of $M_{m,n}\left(\mathbb{R}\right)$. Each point *P* of an image can be defined as an ordered pair P=(i,j)∈P, where P=[[1,m]]×[[1,n]], representing its row and column. On day *t* ∈ [[1, *T*]], an image has a set of *n*_*t*_ visible leaves *L*_*t*_ = {ℓ_*tu*_|*u* ∈ [[1, *n*_*t*_]]}, where $\ell_{tu}\subset P$ is referred to as a leaf segment (or segment for short) and is a connected subset of the image $I_{t}\in M_{m,n}\left(\mathbb{R}\right)$. Over the whole timeline [[1, *T*]], the plant has a set of *N* leaves $L=\left\{L_{v}|v\in \left[\left[1,N\right]\right]\right\}$ indexed by their order of appearance. A particular leaf of the plant is therefore identified by as many occurrences as images in the series, *L*_*v*_ = {ℓ_*tv*_|*t* ∈ [[1, *T*]], ℓ_*tv*_ ∈ *L*_*t*_ ∪ ∅}. A leaf can indeed have the empty set as a segment on certain days if its area is not available, because of overlappings for instance. The index *u* will therefore be reserved to segments, whereas the index *v* will be reserved to leaves. The leaf of rank *v* is the *v*-th to appear. Throughout this work, true segments, considered to be those manually extracted from the images, will be denoted ℓ_*tu*_, while the segments found by the algorithm will be denoted ${\tilde{\ell }}_{tu}$. The same distinction applies to leaves. The problem can therefore be decomposed into two parts:

- Segmentation: on each day *t* ∈ [[1, *T*]], segment the image so as to find as many leaf segments as possible in ${\tilde{L}}_{t}$.
- Tracking: for each leaf of rank *v* ∈ [[1, *N*]], for each day *t* ∈ [[1, *T*]], find if there is an element of ${\tilde{L}}_{t}$ susceptible to belong to ${\tilde{L}}_{v}$ in order to reconstruct the whole history of the *v*-th leaf.

**Figure 2:**
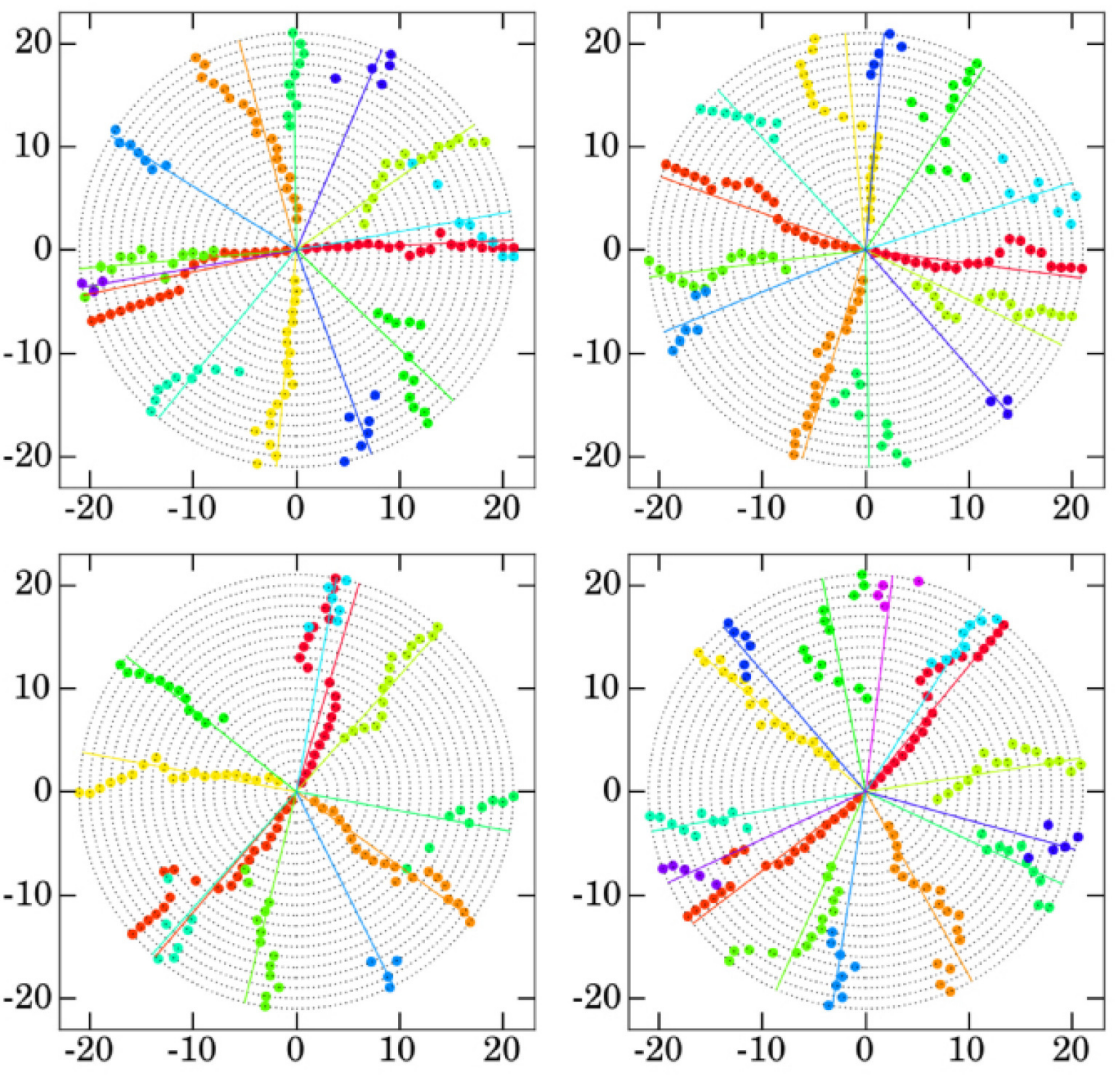
**Evolution of the angles of the different leaves for genotypes Bur (top left), Col (top right), Sha (bottom left), and Tsu (bottom right)**. The angle α of the *v*-th leaf on day *t* is displayed on the circle centered in (0, 0) and of radius *t*, i.e., have coordinates (*t* cos(α), *t* sin(α)). Straight lines indicate the mean angles for each leaf throughout their respective growth. Manually acquired data. 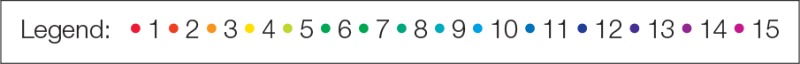

**Figure 3:**
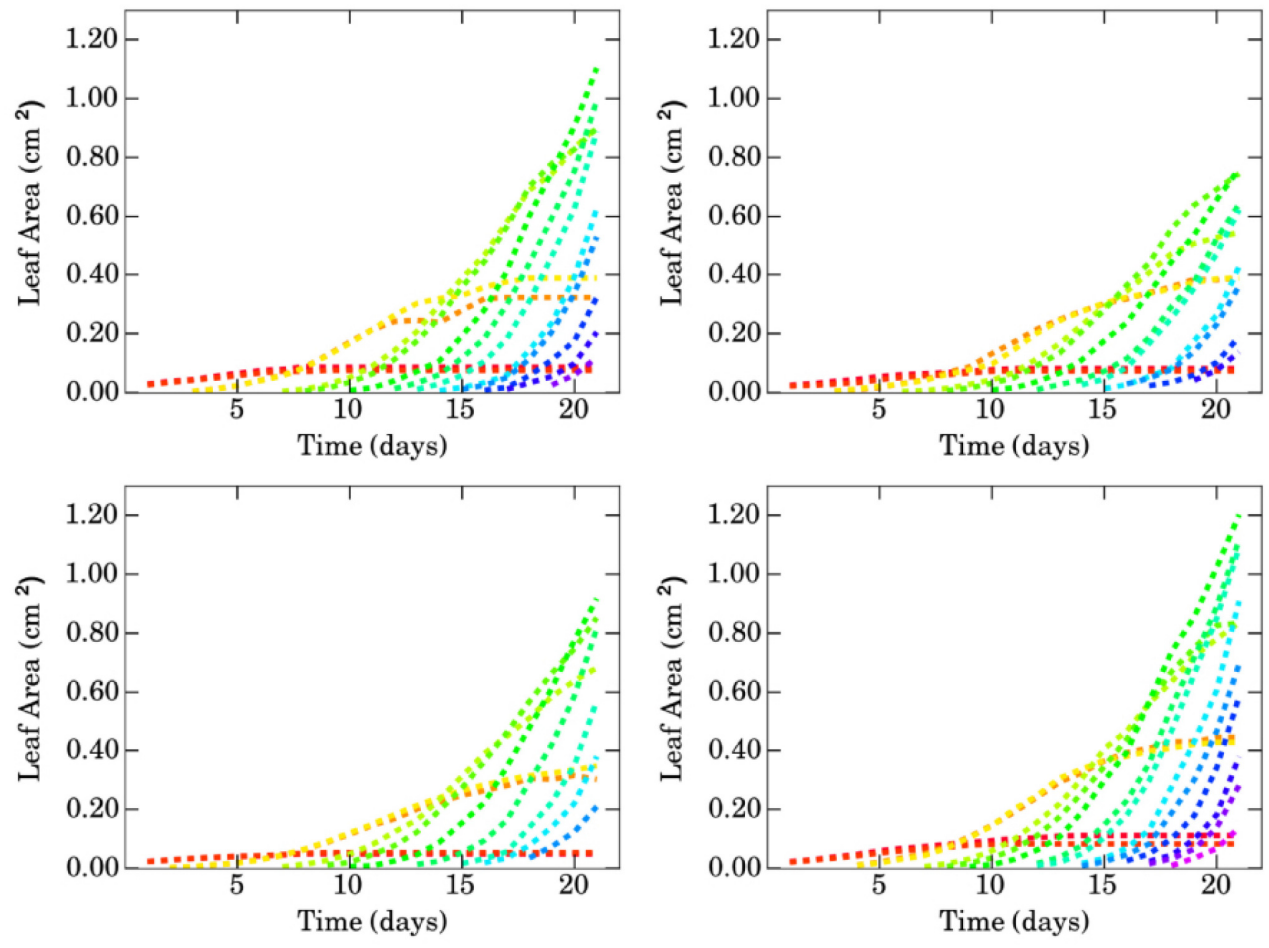
**Evolution of the areas of the different leaves (in *cm*^2^) with respect to time (in days) for genotypes Bur (top left), Col (top right), Sha (bottom left) and Tsu (bottom right)**. Manually acquired data. 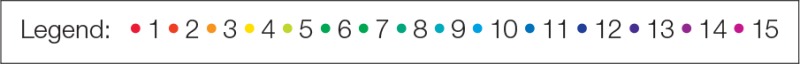

We will denote by C∈P the mass center of the plant. The extremity of a segment ℓ_*tu*_ is defined as the furthest point from the mass center of the plant, i.e., *E*_*tu*_ = arg max_*P*∈ℓ_*tu*__*d*(*P, C*), where *d* is the Euclidean distance. This allows us to measure three variables for a given visible leaf segment ℓ_*tu*_. Let $C{\vec{E}}_{tu}$ be the vector joining the mass center of the plant to the segment extremity, then we define the maximum distance $e_{tu}=||C{\vec{E}}_{tu}|{|}_{1}$ and the maximum angle $d_{tu}=(Oj,C{\vec{E}}_{tu})$ where *Oj* designates the horizontal axis. There are several ways to measure the angle of a segment (other possibilities would be for example the angle defined by the point minimizing the distance to the plant mass center, the average angle of all points or the angle of the mass center of the segment) but this definition is the more stable and robust against overlappings. These two variables yield valuable information about the orientation and the distance of a given leaf throughout plant growth. A third variable is obviously the leaf area, which was manually extracted from the images, potentially reconstructing the shape of the leaves partially hidden by others. It has to be noted that the insertion of the leaf was taken into account when extracting areas. They were manually acquired on all the images using Photoshop CS5, the Ruler tool for angles and distances, which allows easy measurements of distances and angles between two points as well as tab-delimited file export for post-processing, and the Eraser and the Magic Wand to isolate a segment and select all its pixels for the areas. The values obtained for the angles and leaf areas are displayed on Figures 2, 3 respectively for each genotype.

#### 2.2.1. Analysis of angles

As can be seen from Figure 2, the angle of a given leaf is not constant throughout the growth of the plant and there are two main reasons to this: (i) there might be small displacements of the pot from day to day (both in translation and in rotation) and (ii) leaves can be displaced or pushed by some others due to development competition. In most cases, it is easy for a human observer to extract from all the points the occurrences of a given leaf, but sometimes it is very hard not to say impossible to choose between two points. For the Bur genotype, it suffices to consider the trajectories of the 2nd and 6th leaves that create a fork on day 8, or the 10th leaf whose trajectory overrides alternatively that of the 5th leaf and the 1st one. Similar scenarios can be found for the other genotypes.

Let *d*_*tv*_ denote the angle of the *v*-th leaf on day *t*, $d_{v}^{0}$ the angle of the *v*-th leaf on the first day it appeared and ${\bar{d}}_{v}$ the angle of the *v*-th leaf averaged over all the days it exists. On day 1 on the robot, only the first two embryonic leaves (cotyledons) are visible. In fact, 4 leaves are already preformed in *A. thaliana* but they might not be all visible from the very beginning of the image series. The first two leaves grow in opposite directions, i.e. $d_{1}^{0}-d_{2}^{0}\approx 180^{{}^{\circ}}$. The 3rd and 4th leaves (the first true leaves) appear on the same day, more precisely on day 2 for Sha, on day 3 for Bur and Col, and on day 4 for Tsu. Similarly to the first two leaves, they grow in opposite directions such that $d_{3}^{0}-d_{4}^{0}\approx 180^{{}^{\circ}}$. Furthermore, they grow in a direction very close to the bissector of $\left(d_{1}^{0},d_{2}^{0}\right)$, i.e. for $i\in \left\{1,2\right\},j\in \left\{3,4\right\},|d_{i}^{0}-d_{j}^{0}|>40^{{}^{\circ}}$, even though this might not be the case at the end of the growth because of competition, so that for $i\in \left\{1,2\right\},j\in \left\{3,4\right\},|{\bar{d}}_{i}-{\bar{d}}_{j}|>40^{{}^{\circ}}$ does not necessarily hold as can be seen for the Bur, Col and Sha genotypes. By convention and to distinguish between the 1st and 2nd leaves on the one hand and the 3rd and 4th leaves on the other hand, the 1st and 3rd leaves are defined to have the closest averaged angle to that of the 5th leaf, that is $\left|{\bar{d}}_{5}-{\bar{d}}_{1}|\leq |{\bar{d}}_{5}-{\bar{d}}_{2}\right|$ and $\left|{\bar{d}}_{5}-{\bar{d}}_{3}|\leq |{\bar{d}}_{5}-{\bar{d}}_{4}\right|$.

**Figure 4:**
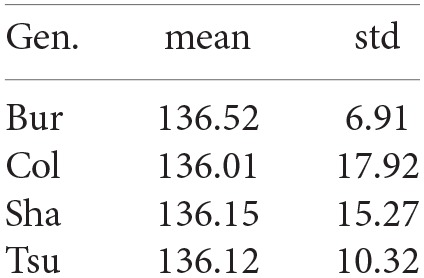
**Mean and standard deviation of the phyllotaxy angle for each genotype**.

The leaves appearing after the 4th one are not preformed and phyllotaxy underlies the direction of their growth. Phyllotaxy is a well-known phenomenon in *A. thaliana* Smith et al. (2006) which drives the growth direction of a leaf based on the growth direction of the leaf previously emerged. More precisely, $|d_{v+1}^{0}-d_{v}^{0}|\approx d_{p}$, where $d_{p}=137.5^{{}^{\circ}}$ is the golden angle. This phenomenon starts from *v* = 4 as it does not affect the preformed leaves and is either clockwise or counter-clockwise for a given plant. However, this orientation cannot be predicted with certainty as it varies among plants: in this case study, it is counter-clockwise for the Bur individual and clockwise for the Col, Sha and Tsu individuals used here. The means and standard deviations of the difference of angles between two consecutive leaves from *v* = 4 are summarized in Figure 4 for the 4 genotypes. This will be used in the classification algorithm to predict the direction of the leaves.

#### 2.2.2. Analysis of areas

From the graphs of the areas on Figure 3, the growth behaviors of the different leaves appear to be similar to those of the angles: the 1st and 2nd leaves have an identical evolution, growing from day 1 to day 10 approximately, then reaching a plateau. The growth is initially linear. Alike, the 3rd and 4th leaves exhibit an identical behavior, appearing on the same day and with a growth curve resembling more a sigmoid than for the first two leaves. By the end of the series, they start to reach a plateau as well. From the 5th leaf, the leaves appear one by one, two leaves never having similar growth curves. The higher the rank of a leaf, the steeper its initial growth so that the area of the *v* + 1-th leaf ends up (or would end up if the series were longer) to exceed that of the *v*-th leaf.

Since there is only one image per day, the phyllochron (that is to say the time interval between the appearance of two successive leaves) cannot be measured exactly, it is however obviously not the same across the different genotypes as can be seen from the number of emerged leaves on day 21: 14 for Bur, 13 for Col, 11 for Sha and 15 for Tsu. The phenotypic differences are well illustrated by, for instance, the area of the 7-th leaf which, on day 21, varies greatly among genotypes: 1.10 cm^2^ for Bur, 0.76 cm^2^ for Col, 0.92 cm^2^ for Sha, 1.20 cm^2^ for Tsu.

### 2.3. Automatic leaf segmentation

The objective of this part is to search for all possible segments of leaves and their corresponding areas on each image of the series. The segmentation problem has been approached in various ways, with recent contributions using ellipsoid leaf-shape models (Aksoy et al., 2015), Gaussian process shape models under a Bayesian approach (Simek and Barnard, 2015) or machine-learning (Pape and Klukas, 2015). The approach used here was inspired from Apelt et al. (2015). Some of the images provided by the Phenoscope software still contained objects from the background. Therefore, connected-segment labeling was first used to discard such objects not belonging to the plant, considered to be the main connected subset. The mass center of the plant is then computed, as it constitutes, once artifacts have been removed, a very good approximation of the stem location from where the leaves grow. Then a Canny edge detection filter (Canny, 1986) is applied to help detect strongly overlapping leaves, before computing the Euclidean distance transform of the plant. The local maxima of this transform are searched, as they are the points the furthest from the background, and are therefore likely to correspond to mass center of leaves. They are hence used as markers for a watershed-based segmentation, which returns a set of connected segments susceptible to be leaves. The image processing operations were all performed using Python 3.4.3, and the library scikit-image 0.12.3.

This first step of the segmentation returns a set of segments ${\tilde{L}}_{t}^{\left(1\right)}=\left\{{\tilde{\ell }}_{tu}^{\left(1\right)}|u\in \left[\left[1,{ñ}_{t}\right]\right]\right\}$, which is different from the true set of segments found manually *L*_*t*_ = {ℓ_*tu*_|*u* ∈ [[1, *n*_*t*_]]}. The main difficulty of segmenting the leaves of a plant classically owes to the fact that some leaves might partially overlap some others, hence leading to segments of the resulting image to be (i) either only parts of a leaf (ii) or several distinct leaves merged. To assess if a segment ${\tilde{\ell }}_{tu}$ can be considered a true leaf segment, we define the ratio:

(2) $$i_{tu}=\frac{A(H(B({\tilde{\ell }}_{tu})))}{A(B({\tilde{\ell }}_{tu}))}$$

where $B:C\to M_{m,n}\left(\left\{0,1\right\}\right)$ transforms a segment into a binary image such that *B*(ℓ)(*i, j*) = δ((*i, j*) ∈ ℓ), $H:M_{m,n}\left(\left\{0,1\right\}\right)\to M_{m,n}\left(\left\{0,1\right\}\right)$ denotes the convex hull operation and $A:M_{m,n}\left(\left\{0,1\right\}\right)\to \mathbb{R}$ gives the area of a segment. If this ratio is greater than a given threshold, the segment is considered to be a leaf, i.e.:

(3) $${\tilde{\ell }}_{tu}\text{ is a leaf segment if }i_{tu}>i_{0}.$$

**Figure 5:**
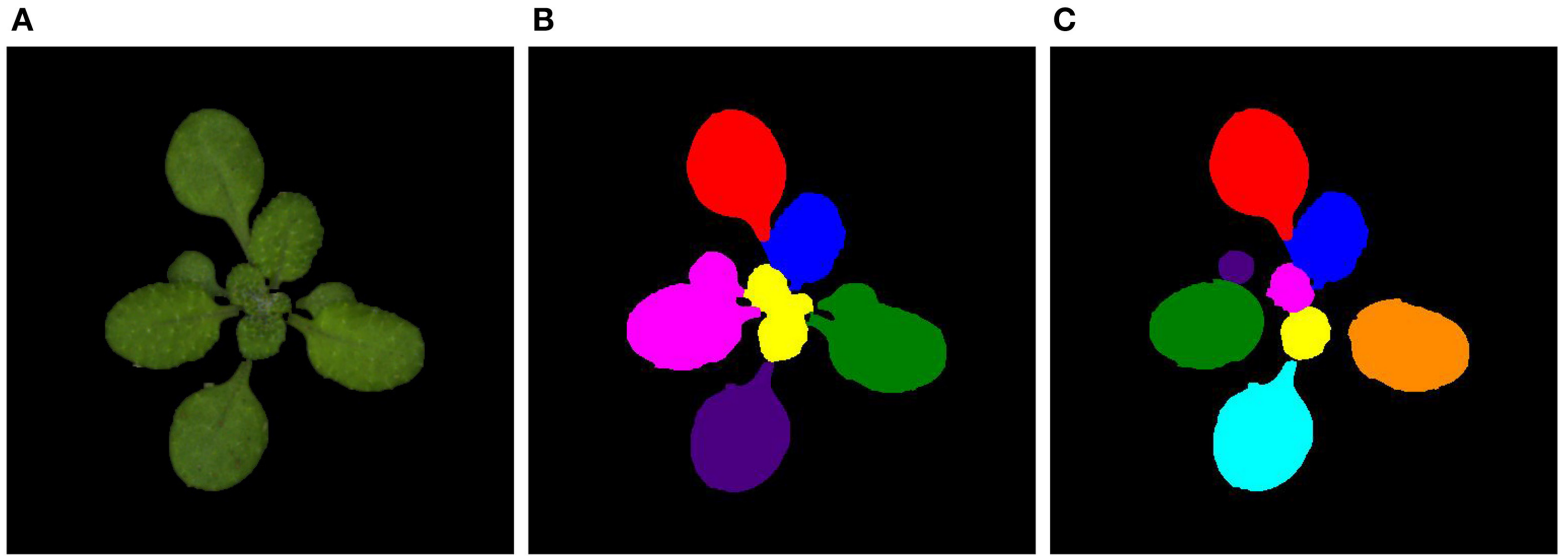
**Example of images output by the Phenoscope and after the two segmentation steps for the Col genotype on day 15. (A)** Output of the Phenoscope. **(B)** After first segmentation. **(C)** After refinement step.

A value *i*_0_ = 0.9 was retained throughout this work. In practice, the result of the first segmentation step is sometimes unable to discriminate between several leaves, grouping them into one single segment. To refine the segmentation, we used a very simple approach. If ${\tilde{\ell }}_{tu}$ is not a leaf segment, the local maxima *M*_*tu*_ of the Euclidean distance transform of ${\tilde{\ell }}_{tu}$ and the points achieving these maxima *I*_*tu*_ are computed:

(4) $$\left\{ \begin{array}{l} I_{tu}=\{P\in {\tilde{\ell }}_{tu}|\;E_{{\tilde{\ell }}_{tu}}(P)\text{ is a local maximum of }E_{{\tilde{\ell }}_{tu}}\},\\ M_{tu}=\{E_{{\tilde{\ell }}_{tu}}(P)|\;P\in I_{tu}\}, \end{array}\right.$$

where $E_{{\tilde{\ell }}_{tu}}=E\left(B\left({\tilde{\ell }}_{tu}\right)\right)$ and $E:M_{m,n}\left(\left\{0,1\right\}\right)\to M_{m,n}\left(\mathbb{R}\right)$ denotes the Euclidean distance transform operation. We then define the greatest two maxima $m_{1}=\max_{m\in M_{tu}}M_{tu},m_{2}=\max_{m\neq m_{1}}M_{tu}$ and their corresponding coordinates *P*_1_ and *P*_2_, and two ellipses *E*_1_ and *E*_2_ with respective centers *P*_1_ and *P*_2_, minor semi-axes *a*_1_ and *a*_2_ and major semi-axes *b*_1_ and *b*_2_, where:

(5) $$a_{k}=\phi \;\underset{P\in (CE_{k})}{\min}d(P,E_{k}),\;b_{k}=\phi \;\underset{P|(PE_{k})⊥(CE_{k})}{\min}d(P,E_{k}).$$

with ϕ = 1.05 to embrace the whole leaf segment. Two new segments are thus computed, ${\tilde{\ell }}_{tu}^{\cap }={\tilde{\ell }}_{tu}\cap E_{1}$ and ${\tilde{\ell }}_{tu}^{\backslash }={\tilde{\ell }}_{tu}\backslash {\tilde{\ell }}_{tu}^{\cap }$ and tested to be leaves again in a recursive manner. Results of this refinement step are illustrated on Figure 5.

### 2.4. Leaf tracking

The segmentation step returns $\tilde{ℒ}$ such that:

(6) $$\left\{ \begin{array}{l} \tilde{ℒ}=\{{\tilde{L}}_{t}|t\in [[1,T]]\},\\ {\tilde{L}}_{t}=\{{\tilde{\ell }}_{tu}|u\in [[1,{\tilde{n}}_{t}]]\}, \end{array}\right.$$

The true set of leaves L is such that:

(7) $$\left\{ \begin{array}{l} ℒ=\{L_{t}|t\in \;[[1,T]]\},\\ L_{t}=\{\ell_{tu}|u\in \;[[1,n_{t}]]\}, \end{array}\right.$$

or equivalently:

(8) $$\left\{ \begin{array}{l} ℒ=\{L_{v}|v\in [[1,N]]\},\\ L_{v}=\{\ell_{tv}|t\in [[1,T]],\ell_{tv}\in L_{t}\cup \emptyset \}. \end{array}\right.$$

The objective of the tracking step is to assign each segment ${\tilde{\ell }}_{tu}$ found during the segmentation step to a leaf of a given rank so that $\tilde{L}=\{{\tilde{L}}_{v}|v\in [[1,N]]\}$ with ${\tilde{L}}_{v}=\{{\tilde{\ell }}_{tv}|t\in [[1,T]],{\tilde{\ell }}_{tv}\in {\tilde{L}}_{t}\cup \emptyset \}$ be as close as L. The data analysis helped us to better understand the growth dynamics of each leaf. The tracking of a given leaf is based on its direction, its maximum distance and its area. To find the first occurrence of a leaf, only its direction and the day of possible first appearance are given. Let us recall that the first two leaves (cotyledons) always appear on day 1. Once an occurrence of the leaf has been found, it is searched on the following days according to this strategy: given a leaf of rank *v* whose *k* first segments have been tracked, ${\tilde{L}}_{v}=\{{\tilde{\ell }}_{tv}|t\in [[1,k]]\}$, the aim is to find the segment on day *k* + 1 the most probable to belong to the leaf of rank *v*. For each leaf ${\tilde{l}}_{k+1,u}\in {\tilde{L}}_{k+1}$, a score *s*_*kuv*_ is computed as:

(9) $$s_{kuv}=\gamma_{d}\;s_{kuv}^{d}+\gamma_{e}\;s_{kuv}^{e}+\gamma_{a}\;s_{kuv}^{a}$$

where the superscripts *d*, *e* and *a* stand for direction, extremity and area respectively, $\left(\gamma_{d},\gamma_{e},\gamma_{a}\right)\in {\left({\mathbb{R}}^{+}\right)}^{3}$ allows to weigh the different scores and:

(10) $$\left\{ \begin{array}{l} s_{kuv}^{d}=2\delta d\;f_{U}(d_{k+1,u},d_{kv}-\delta d,d_{kv}+\delta d)\\ s_{kuv}^{e}=f_{N}(e_{k\;+\;1,u},\mu^{e},{\left(\sigma^{e}\right)}^{2})/\|f_{N}(\cdot ,\mu^{e},{\left(\sigma^{e}\right)}^{2}){\|}_{\infty }\\ s_{kuv}^{a}=f_{N}(a_{k\;+\;1,u},\mu^{a},{\left(\sigma^{a}\right)}^{2})/\|f_{N}(\cdot ,\mu^{a},{\left(\sigma^{a}\right)}^{2}){\|}_{\infty } \end{array}\right.$$

where $f_{U}$ and $f_{N}$ are the probability density functions of the uniform and normal distributions.

The *d*-score $s_{kuv}^{d}$ favors the leaves that grow in the same direction of the last leaf segment ${\tilde{l}}_{k,v}$, with a tolerance of δ*d* to account for pot rotations or competition of the growing leaves, as explained in Section 2.2.1. In practice, δ*d* = 30°. As can be seen from Figure 2, it does not seem useful to take into account the directions of the leaves ${\tilde{\ell }}_{tu}$ for *t* < *k*: the rotations being seemingly unpredictable, neither averaging nor interpolating seem of any help, and the last value is the one carrying the most information.

Data analysis of the areas and the distances of the extremities from the mass center showed that their dynamics were sigmoid-like, which is why the means and standard deviations in the *a*-score and the *e*-score are obtained, whenever possible, by fitting a sigmoid using the last 4 segments of the leaf. Since segments might not be found on all images for a given leaf, the last 4 segments do not necessarily represent the last 4 days. More precisely, let {*t*_*k*+1−*i*_|*i* ∈ [[1, 4]]} denote the days on which were registered the last 4 segments of the *v*-th leaf and let *s* be such that:

(11) $$\begin{array}{l} s_{\kappa }(x)=y_{1}+\frac{y_{0}}{1+\exp(-k\;(x-x_{0}))},\\ \;with\;\kappa =(k,x_{0},y_{0},y_{1}) \end{array}$$

For the prediction of the expected extremity on day *k* + 1, we define:

(12) $$\left\{ \begin{array}{l} {\hat{\kappa }}^{e}=\text{arg min}\sum_{i=1}^{4}\|s_{\kappa }(t_{k+1-i})-e_{t_{k+1-i},v}{\|}_{2}^{2},\\ \mu^{e}=s_{{\hat{\kappa }}^{e}}(k+1). \end{array}\right.$$

All the same, for the prediction of the expected area:

(13) $$\left\{ \begin{array}{l} {\hat{\kappa }}^{a}=\text{arg min}\sum_{i=1}^{4}\|s_{\kappa }(t_{k+1-i})-a_{t_{k+1-i},v}{\|}_{2}^{2},\\ \mu^{a}=s_{{\hat{\kappa }}^{a}}(k+1). \end{array}\right.$$

The standard deviations use only the last available value, $\sigma^{a}=2\;a_{kv}$ and $\sigma^{e}=e_{kv}/2$. When less than 4 occurrences are available, μ_*e*_ = 1.2 *e*_*kv*_ and μ_*a*_ = 2 *a*_*kv*_. Let û be the segment index with greatest score, û = arg max_*u*_ *s*_*kuv*_ and ŝ = *s*_*kûv*_ the corresponding score. The safety score *s*_0_ is defined as a minimum score to achieve to be considered a segment: hence, if ŝ > *s*_0_, the candidate segment ${\tilde{\ell }}_{k+1,û}$ achieving this best score is considered to be the segment of the leaf of rank *v* on day *k* + 1, and ${\tilde{L}}_{v}:={\tilde{L}}_{v}\cup {\tilde{\ell }}_{tu}$. In practice, typical values of the score weights and the safety score would be (γ_*d*_, γ_*e*_, γ_*a*_) = (10, 1, 1) and *s*_0_ = 11, thereby prioritizing the orientation of a leaf over its length and area.

In order to take advantage of the phyllotaxy, the preformed leaves are first classified, the 1st and 2nd leaves first, in opposite directions, then the 3rd and 4th (the first 2 true leaves), in opposite directions and perpendicular to the first two. Classifying the 5th leaf will then yield the directions of the next leaves, which are the hardest to classify. The whole tracking strategy is summarized on Algorithm 1.

**Algorithm 1:** Classification strategy (leaves 1 and 2 are the cotyledons).

1: Track leaf 1 (randomly out of the two leaves found on day 1) for all days.
2: Look for leaf 2 in direction *d*_1_ + 180° and track it.
3: Look for leaf 3 in directions such that |*d*_3_ − *d*_1_| > 60° and |*d*_3_ − *d*_2_| > 60° and track it.
4: Look for leaf 4 in direction *d*_3_ + 180° and track it.
5: Track the 5th leaf to appear, whatever its growth direction.
6: Shuffle leaves 1 and 2 so that leaf 1 is closest to leaf 5 (convention).
7: Shuffle leaves 3 and 4 so that leaf 3 is closest to leaf 5 (beginning of phyllotaxy).
8: **for** *j* ≥ 1 **do**
9: Look for leaf 5 + *j* in direction *d*_5_+*j* sign(*d*_5_−*d*_4_) *d*_*p*_ and track it.
10: **end for**

In the next section, we will present a simple growth model for the rosette stage of *A. thaliana* which will be parameterized for each genotype using the data obtained through the image analysis methodology.

### 2.5. An organ-scale plant model

The GreenLab model (Yan et al., 2004) is a typical functional-structural model in the sense that it combines the description of plant architectural development and ecophysiological functioning. A version has been developed for the full cycle of *A. thaliana* growth in Christophe et al. (2008). Basically, a developmental submodel predicts organ appearances while source-sink dynamics is simultaneously described: biomass production is computed via radiation interception by leaf area and the produced biomass is allocated between all growing organs according to individual sink strengths. Individual leaf areas are then deduced from leaf masses. In our study, only the rosette stage of Arabidopsis growth is considered, which particularly simplifies the organogenesis submodel and the number of competing sinks. Moreover, at this stage, the senescence process has not started yet.

#### 2.5.1. Organogenesis

As detailed in section 2.2.1, leaves first appear in pairs (1st and 2nd leaves together, then 3rd and 4th leaves) before the following ones start appearing rhythmically. The time span between the appearances of two successive leaves is called phyllochron Wilhelm and McMaster (1995). It is mostly driven by the thermal time, that is to say the accumulated growing degree days. However, in controlled and constant thermal conditions as in the Phenoscope, it amounts to considering the calendar time as the main driver of organogenesis. For a better understanding of the source-sink dynamics in this first stage of study, we consider that the leaf appearance times of the first 4 leaves are known, whereas those of the subsequent leaves are such that their difference is always the phyllochron ϕ (in h).

#### 2.5.2. Biomass production

Plant growth starts when the thermal time becomes sufficient (germination). At this time, the biomass is supposed to be that of the seed *q*_0_. To take into account the photoperiod and the differences in temperature between day and night, the time step is taken to be the hour. Once growth has started, the biomass produced at time step *t* is given by

(14) $$q(t)=r(t)\;\mu \;s\left(1-\exp\left(-\frac{k}{s\;e}\sum_{v\in [[1,n(t)]]}Q_{v}(t)\right)\right)$$

where *r*(*t*) is the photosynthetically active radiation (in MJ cm^2^ h^−1^), μ is the radiation use efficiency (in g MJ^−1^) and *s* is related to the projected area of the plant (in cm^2^), *k* is the Beer–Lambert law coefficient of light extinction (dimensionless), *e* is the leaf mass per surface area (in g cm^−2^), *n*(*t*) is the number of leaves of the plant at time step *t* and *Q*_*v*_(*t*) is the biomass of the *v*-th leaf (in g).

#### 2.5.3. Biomass allocation

The pool of produced biomass is then allocated to the different organs of the plant. In the present case, that is to say the rosette stage, only the leaves are considered. The biomasses allocated to the different leaves are proportional to their respective demands, or sink strengths, which are functions of their expansion stage, i.e., the thermal time since appearance. In previous Greenlab models, Beta distributions were used for the sink functions. This is not possible in the present case since (i) the expansion period of the leaves is not known and (ii) over the period of time of interest, some of the leaves still grow significantly. Instead, lognormal distributions were used instead as they allow for a similar growth dynamics with an ever ongoing growth. As was suggested by the analysis of the areas of the different leaves, two different functions were used for the first 4 (preformed) leaves on the one hand and the leaves with rank higher than 5 on the other, the demand of the *v*-th leaf hence being:

(15) $$d_{v}(t)\text{​}=\text{​}\{\begin{array}{l} \text{​}f_{\log\;N}(\tau (t)-\tau_{v},\mu_{1},\sigma_{1}^{2})\text{ ​}/\text{​ }\|f_{\log\;N}(\cdot ,\mu_{1},\sigma_{1}^{2}){\|}_{\infty }\text{ if }v\leq 4,\\ \rho \;f_{\log\;N}(\tau (t)-\tau_{v},\mu_{2},\sigma_{2}^{2})\;/\;\|f_{\log\;N}(\cdot ,\mu_{2},\sigma_{2}^{2}){\|}_{\infty }\text{ if }v>4. \end{array}$$

Dividing by the uniform norms ensures that *d*_*v*_(*t*) ∈ [0, 1] for all *v* ∈ ℕ^⋆^ and *t* ∈ ℕ^⋆^, in order to avoid a bias resulting from the variation of the function maximum with their parameters, though a coefficient ρ ≈ 1 allows for a different intensity of the two different kinds of leaves. Here, $f_{\log N}$ is the probability density functions of the lognormal law, τ(*t*) is the thermal time at time step *t* and τ_*v*_ is the accumulated thermal time of the *v*-th leaf since its emergence (both in °C h). $\left(\mu_{1},\sigma_{1}^{2}\right)$ and $\left(\mu_{2},\sigma_{2}^{2}\right)$ are the parameters of the lognormal distributions for the preformed leaves and those with rank higher than 5 respectively. The biomass allocated to a leaf is then the relative demand of the available produced biomass:

(16) $$q_{v}(t)=\frac{d_{v}(t)}{\sum_{w\in [[1,n(t)]]}d_{w}(t)}q(t),$$

which allows to update the cumulated biomasses:

(17) $$Q_{v}(t)\;=Q_{v}(t-1)\;+q_{v}(t),$$

and compute the related leaf areas, with *e* the leaf mass per surface area:

(18) $$A_{v}(t)=\frac{1}{e}Q_{v}(t).$$

## 3. Results

### 3.1. Estimation of leaf areas through image processing

**Figure 6:**
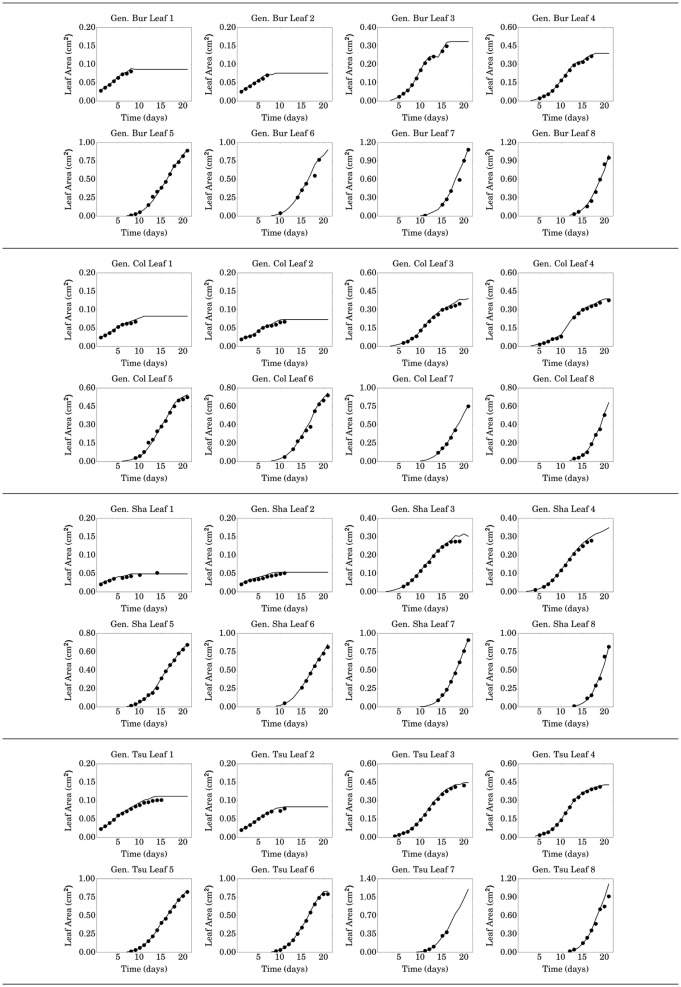
**Results obtained manually (continuous line) and via the segmentation-classification approach (filled circles) for the first 8 leaves of the 4 genotypes**.

The results for the first 8 leaves (including the 2 embryonic leaves) of the 4 different genotypes are summarized on Figure 6, where the true results obtained with manual segmentation of the images, displayed as a continuous line, are compared to the results of the segmentation-tracking algorithm, displayed as filled circles. It has to be noted that the manual extraction of the leaf areas partly took into account the petiole, which is why the algorithmic results are on average lower than the manual ones.

For the first two leaves (the cotyledons), no segments are found from day 15 (or even before sometimes), as these leaves are rapidly completely masked by younger leaves. On some days, no segments were found: for instance, for the 6th leaf of the Bur genotype, the first segment is found on day 10 but from day 11 up to day 13, no segment is found. Such a situation arises because of overlappings and (i) either the segmentation step is unable to identify segments at all for this leaf on these days or (ii) segments are found but they do not achieve a sufficient score to be considered as belonging to this leaf. In the latter case, it is preferable to discard these segments so as not to introduce too noisy data. Another scenario for missing data occurs when the growth curve of a leaf displays an unusual shape as is the case of the 4th leaf of the Col genotype, with a sharp increase after day 10. The predicted area obtained by fitting a sigmoid using the last 4 segments of this leaf is therefore too low compared to that of the segment yielding an insufficient overall score to be accepted. In any case, this does not, fortunately, prevent the algorithm to find new segments on future days: starting from day 14 for the 6th leaf of the Bur genotype, or from day 13 for the 4th leaf of the Col genotype, therefore highlighting the robustness of the method toward missing data.

The evaluated leaf areas will be used to estimate the parameters of the GreenLab functional-structural plant model presented in the next section, by model inversion. For a given leaf, only conserving segments in which there is a very high level of confidence might seem overly cautious. However, since the architecture is in itself representative of the whole plant functioning, there is a lot of redundant information contained in leaf area at close enough times. The daily image acquisition of the Phenoscope is thus in excess with respect to the time scale of *A. thaliana* source-sink dynamics. Overall, all the accepted points have a very low error compared to the true values and the amount of very precise data this method yields for a given plant (more than 60 values for each genotype) will prove sufficient for an accurate parameter estimation of the model.

**Figure 7:**
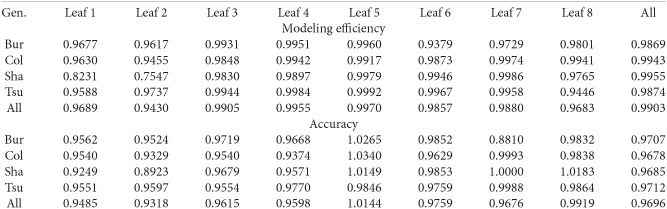
**Modeling efficiency and accuracy for the different leaves and genotypes**.

If *y* = (*y*_*i*_)_1≤*i*≤*N*_ denotes the true dataset to estimate and ỹ = (ỹ_*i*_)_1≤*i*≤*N*_ the corresponding estimates, the modeling efficiency ϵ Wallach (2006) (also called the coefficient of determination) and the accuracy α with which the leaf area has been estimated can be calculated as:

(19) $$\left\{ \begin{array}{l} \epsilon =1-\sum_{i=1}^{N}{\left(y_{i}-{\tilde{y}}_{i}\right)}^{2}\;/\;\sum_{i=1}^{N}{\left(y_{i}-\bar{y}\right)}^{2}\\ \alpha =\frac{1}{N}\sum_{i=1}^{N}\left|\frac{{\tilde{y}}_{i}}{y_{i}}\right| \end{array}\right.$$

where ȳ is the mean value of *y*. The higher the modeling efficiency and the closer to one the accuracy, the better. Different such criteria were computed for completeness: one per individual leaf to account for the fact that the mean leaf area can be of different orders of magnitudes for different leaves, one per genotype for the different leaves, one per leaf for the different genotype and a global one taking into account all the data available. The results are displayed in Figure 7. All the modeling efficiencies are greater than 0.93 except for the first two leaves of the Sha genotype, highlighting the overall excellent quality of the data extracted from the Phenoscope images for the leaf area. It has to be noted that the accuracy is most often below 1, which means that the results are a bit underestimated on average. This is mostly due to taking into account the insertion of the leaf when extracting areas manually.

### 3.2. Model parameterization

**Figure 8:**
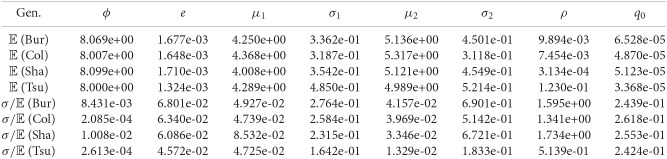
**Estimated parameters for the 4 genotypes (mean values and relative errors obtained by dividing the standard errors by the means)**.

**Figure 9:**
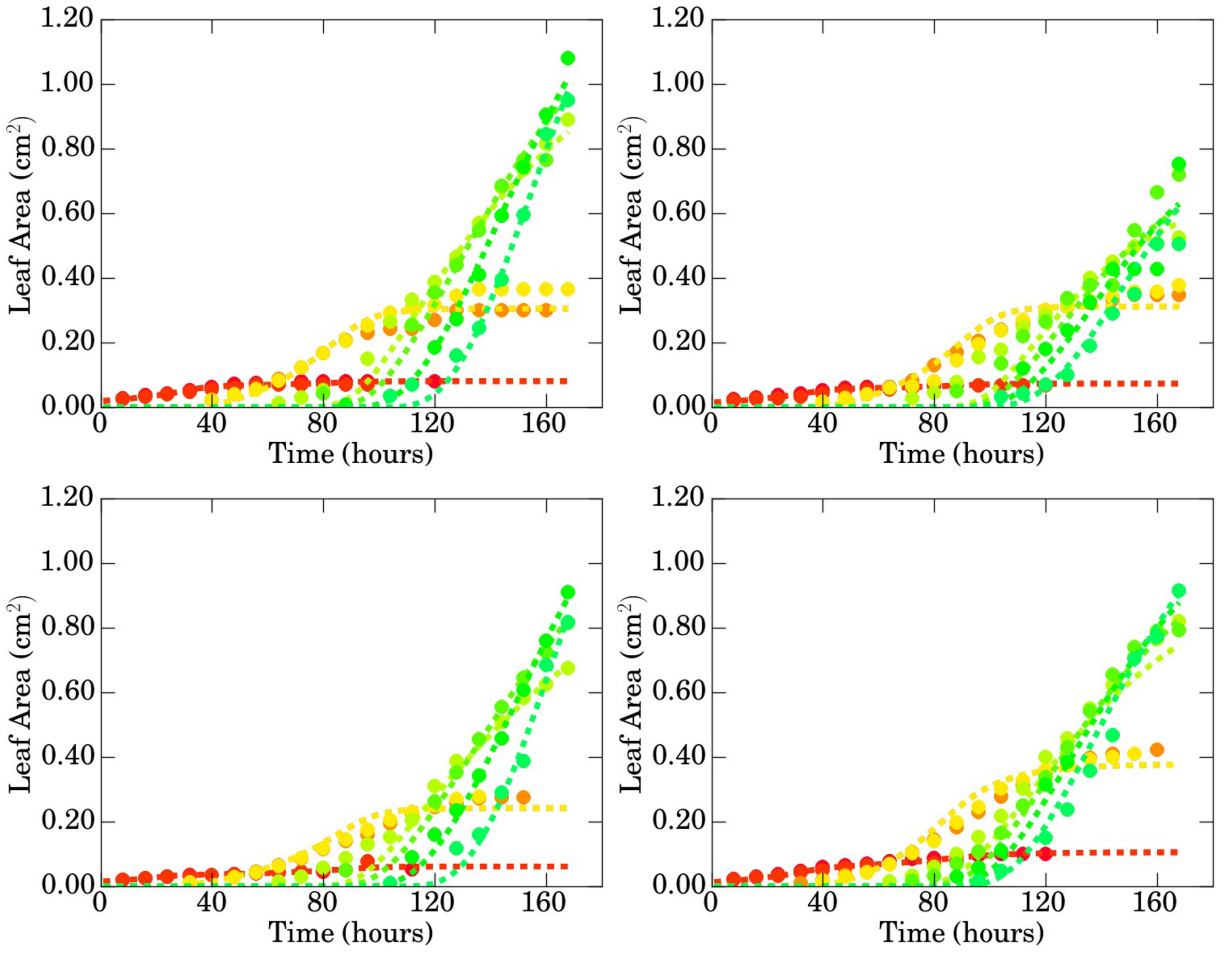
**Comparison of the areas of the different leaves (in *cm*^2^) with respect to time (in hours) for genotypes Bur (top left), Col (top right), Sha (bottom left), and Tsu (bottom right)**. Data obtained through image processing is represented by filled circles while predictions of the calibrated model are represented by dashed lines. 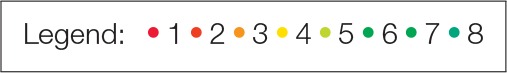

**Figure 10:**
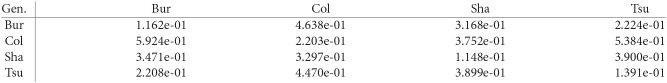
**Matrix of the normalized root mean square error: the component (*i, j*) is the normalized RMSE between the simulated data of genotype *i* with the estimated set of parameters vs. the experimental data of genotype *j***. The lowest values are on the diagonal, confirming the capability of the model to correctly distinguish between genotypes.

The leaf areas obtained through image processing are used to calibrate the model: for each genotype, such data can be seen as a vector of vector of areas (one vector of area per leaf) with possibly missing data. The observations of the model are then set accordingly for each leaf and both the simulation and observation data can be flattened into a single vector. Such a parameterization of the GreenLab model using a generalized least squares procedure with multiplicative noise has been extensively described in Cournède, P.-H. et al. (2011). Here the parameter subset θ_*e*_ = (ϕ, *e*, μ_1_, σ_1_, μ_2_, σ_2_, ρ, *q*_0_) ⊂ θ was estimated. The results of the estimated parameters are given in Figure 8 for the different genotypes. The estimation accuracy given by the standard errors are satisfactory, and the estimated parameter set differs substantially from one genotype to another. In Figure 9, we illustrate the fitting of the leaf areas observed vs. those predicted by the model, with a proper adequation. To emphasize the capability of the whole methodology comprising image analysis and model calibration, the normalized root mean square error (NRMSE) (Wallach, 2006) between the data obtained by simulation with the estimated set of parameters for genotype *i* vs. the experimental data for genotype *j* were computed. For the experimental data of a given genotype *i*, the lowest NRMSE was always found for the data simulated using the estimated set of parameters for this genotype, confirming the capacity of the model to correctly differentiate between genotypes. These results are displayed in Figure 10. Even if some further statistical analysis beyond the scope of this paper should be conducted to analyze the differences between genotypes, these encouraging results pave the way for the implementation of the methodology at larger scales with the hope of new tools for the analysis of genotypic differences.

## 4. Discussion

The Phenoscope, a high-throughput phenotyping platform, provided images of *A. thaliana* for different genotypes grown in the same environmental conditions. The behavior similarities and differences of some variables on these images for the different genotypes, such as orientation, distance from the mass center or area of the different leaves, were highlighted. They helped build a two-step algorithm for leaf segmentation and tracking, allowing to reconstruct the whole history of the different leaves. The comparison of the results obtained for leaf area with the true results, extracted from the images manually, showed that this procedure yields numerous and very precise data. Having obtained such data for the different leaves of the plant makes it possible to design an organ-scale plant model, based on the existing GreenLab model, and better understand the dynamics of leaf growth regulation and disentangle the effects of leaf growth and leaf emission rates (Tisné et al., 2010). The experimental data obtained with the help of the segmentation-tracking algorithm was used to parameterize the model for the different genotypes using a generalized least squares estimator. Primary results show that the optimal estimated parameter set is different for the 4 genotypes.

This work represents the first step of a study of genotypic differentiation within *A. thaliana*, and further investigation is still needed on several fronts. First, even though the results obtained on the limited (though genetically diverse Simon et al., 2012; McKhann et al., 2004) sample of Bur, Col, Sha, and Tsu are so far very encouraging, the image processing routine must be tested on many more image series that the Phenoscope can provide (Tisné et al., 2013), for several dozens of genotypes with different individuals within each genotype to allow statistical testing of genotypic differentiation (Reymond et al., 2003). Environmental conditions such as altitude, rainfall, soil nutrient content, etc. influence leaf size (McDonald et al., 2003; Scoffoni et al., 2011), but environmental influences, in particular light, can also influence leaf shape (Tsukaya, 2005), even though the underlying mechanisms are not yet fully understood. Therefore, it is all the more important to test whether more sophisticated shape models (Herdiyeni et al., 2015; Simek and Barnard, 2015) could replace our relatively simple ellipse-based one and help estimate leaf areas more precisely or even increase the rate of leaf detection. Likewise, regarding leaf tracking, a promising approach would be to develop an iterative method in which, after a first step based on our original approach, the parameterized model thus obtained could be used instead of the sigmoid extrapolation to predict individual leaf areas at the next time step. In our original approach, there were many cases in which leaf segments were discarded in the tracking step due to a low score resulting from a bad performance of the sigmoid growth function (for example because there were not enough segments of the corresponding leaf in the previous steps). Therefore, we expect that model-based predictions could significantly improve the number of detected segments for each leaf.

As underlined above, we used a very cautious approach and discarded data for which we did not get a very high level of confidence. Indeed, owing to the redundant information contained in the sequence of plant architectural descriptions, already pointed out in Godin et al. (1999), we expected that it would not impact the quality of model identification. Our results seem to support this hypothesis. However, it would also be interesting to study more carefully the impact of the safety score *s*_0_ on the rate of detection (both in terms of true positive rate and false positive rate), but also on the quality of model estimation. There are two aspects to this last question: how does the model estimation handle false data, and how does missing data impact the uncertainty in parameter estimates. Obviously, every refinement of the method that could help reduce such uncertainty will be profitable. Similarly, designing new data collection protocols for the Phenoscope that are adapted to the model identification objective could also be considered (for example by measuring plant organ masses, using several individuals of the same genotype to take into account inter-individual variability, etc.). Optimal experimental design methodology could help us for this purpose (Pieruschka and Poorter, 2012; Craufurd et al., 2013).

More generally, the statistical evaluation of model parameter estimation is a crucial issue in order to properly assess whether a model has the capacity to differentiate between genotypes. When can two genotypes be considered to have significantly different parameters? Can a parameter be considered stable in a family of genotypes or environments? A proper statistical approach has to be implemented for this purpose. In particular, to account for the interindividual variability, the use of statistical models such as multilevel/hierarchical models (Gelman and Hill, 2007) or mixture models (Tatarinova and Schumitzky, 2015), should be investigated. A methodology involving hierarchical mixed effects models and testing of variance components is proposed in Baey et al., (in preparation) and should be used in order to clearly identify which parameters can be considered constant or varying among the population of genotypes. Finally, being able to identify plant model parameters varying among genotypes from high-throughput phenotyping data is the first step toward the integration of genetic control into plant models (Baldazzi et al., 2016; Xu and Buck-Sorlin, 2016) via QTL or association mapping (Myles et al., 2009).

## Author contributions

All authors listed, have made substantial, direct and intellectual contribution to the work, and approved it for publication.

## Funding

This work was supported by CentraleSupélec, University of Paris-Saclay and the National Institute of Agricultural Research.

### Conflict of interest statement

The authors declare that the research was conducted in the absence of any commercial or financial relationships that could be construed as a potential conflict of interest.
